# Application of multi-omic features clustering and pathway enrichment to clarify the impact of vitamin B2 supplementation on broiler caeca microbiome

**DOI:** 10.3389/fmicb.2023.1264361

**Published:** 2023-09-28

**Authors:** Carlo Mengucci, Simone Rampelli, Gianfranco Picone, Alex Lucchi, Gilberto Litta, Elena Biagi, Marco Candela, Gerardo Manfreda, Patrizia Brigidi, Francesco Capozzi, Alessandra De Cesare

**Affiliations:** ^1^Department of Agricultural and Food Sciences, University of Bologna, Cesena, Italy; ^2^Department of Pharmacy and Biotechnology, University of Bologna, Bologna, Italy; ^3^DSM Nutritional Product, Kaiseraugst, Switzerland; ^4^Department of Civil, Chemical, Environmental, and Materials Engineering, University of Bologna, Bologna, Italy; ^5^Department of Medical and Surgical Sciences, University of Bologna, Bologna, Italy; ^6^Department of Veterinary Medical Sciences, University of Bologna, Ozzano Emilia (BO), Italy

**Keywords:** vitamin B2, microbiome, pathway enrichment, feature clustering, broilers

## Abstract

**Background:**

The results of omic methodologies are often reported as separate datasets. In this study we applied for the first time multi-omic features clustering and pathway enrichment to clarify the biological impact of vitamin B2 supplementation on broiler caeca microbiome.

**Methods:**

The caeca contents of broilers fed +50 and +100 mg/kg vitamin B2 were analyzed by shotgun metagenomic and metabolomic. Latent variables extracted from NMR spectra, as well as taxonomic and functional features profiled from metagenomes, were integrated to characterize the effect of vitamin B2 in modulating caeca microbiome. A pathway-based network was obtained by mapping the observed input genes and compounds, highlighting connected strands of metabolic ways through pathway-enrichment analysis.

**Results:**

At day 14, the taxonomic, functional and metabolomic features in the caeca of tested broilers showed some degree of separation between control and treated groups, becoming fully clear at 28 days and persisting up to 42 days. In the caeca of birds belonging to the control group *Alistipes* spp. was the signature species, while the signature species in the caeca of broilers fed +50 and +100 mg/kg vitamin B2 were *Bacteroides fragilis* and *Lactobacillus crispatus*, *Lactobacillus reuteri*, *Ruminococcus torques*, *Subdoligranum* spp., respectively. The pathway enrichment analysis highlighted that the specific biochemical pathways enhanced by the supplementations of vitamin B2 were N-Formyl-L-aspartate amidohydrolase, producing Aspartate and Formate; L-Alanine:2-oxoglutarate amino transferase, supporting the conversion of L-Alanine and 2-Oxoglutarate in Pyruvate and L-Glutamate; 1D-myo-inositol 1/4 phosphate phosphohydrolase, converting Inositol 1/4-phosphate and water in myo-Inositol and Orthophosphate. The results of this study demonstrated that the caeca of birds fed +50 and + 100 mg/kg were those characterized by taxonomic groups more beneficial to the host and with a higher concentration of myo-inositol, formic acid, amino acids and pyruvate involved in glycolysis and amino acid biosynthesis.

**Conclusion:**

In this study we demonstrated how to perform multi-omic features integration to describe the biochemical mechanisms enhanced by the supplementation of different concentrations of vitamin B2 in the poultry diet. The relationship between vitamin B2 supplementation and myo-inositol production was highlighted in our study for the first time.

## 1. Introduction

Vitamins are micronutrients with important physiological effects on various biological responses, including host immunity, and resulting in a number of health benefits (Suwannasom et al., 2020). Vitamins are synthesized by bacteria, yeasts, and plants, but chickens must obtain vitamins from their diet or rely on their synthesis by commensal bacteria in the gastrointestinal tract (Yoshii et al., 2019). Among vitamins, vitamin B is water soluble and not stored by the body, meaning that any excess is excreted in the urine. In chickens, vitamin B2 (riboflavin) deficiencies result in nervous malformations, footpad dermatitis and ‘curled-toe paralysis’ (Cai et al., 2006; Shepherd and Fairchild, 2010). The dose of vitamin B2 supplementation recommended in the chicken diet for more than 50 years has been 3.6 mg/kg feed (National Research Council, 1994). However, Olkowski and Classen (1998), investigated the metabolic requirement of vitamin B2 by different organs and the impact of different vitamin B2 concentrations in the feed on zootechnical parameters of fast-growing chickens, concluding that at least 5 mg/kg of riboflavin should be included in the diet. Recently breeding companies suggested doses even higher (e.g., 8.6 mg/kg starter feed for fast-growing genotypes) (Aviagen, 2019).

From a chemical point of view, riboflavin is 7,8-dimethyl-10-ribityl-isoalloxazine consisting of a flavin isoalloxazine ring bound to a sugar side chain, ribitol (Dym and Eisenberg, 2001). Riboflavin plays a role in a variety of metabolic pathways, serving primarily as an integral component of its crucial biologically active forms, the flavocoenzymes flavin adenine dinucleotide (FAD) and flavin mononucleotide (FMN). These flavocoenzymes ensure the functionality of numerous flavoproteins, including dehydrogenases, oxidases, monooxygenases, and reductases, playing pivotal roles in mitochondrial electron transport chain, β-oxidation of fatty acids, redox homeostasis, citric acid cycle, branched-chain amino acid catabolism, chromatin remodeling, DNA repair, protein folding, and apoptosis (Balasubramaniam and Yaplito-Lee, 2020). Vitamin B2 deficiency suppresses the activity of acyl-CoA dehydrogenases, involved in the oxidation of fatty acids to generate acetyl-CoA, which is used by mitochondria to produce ATP *via* the tricarboxylic acid (TCA) cycle (or the Krebs cycle) (Yoshii et al., 2019). Fatty acid oxidization is involved in the activation, differentiation, and proliferation of immune cells through the generation of acetyl-CoA and its entry into TCA cycle (Almeida et al., 2016). Moreover, vitamin B2 is associated with reactive oxygen species generation in immune cells through the priming of NADPH oxidase 2 (Schramm et al., 2014); those reactive oxygen species are important effector and signaling molecules in inflammation and immunity (Yoshii et al., 2019).

As in humans, in the chicken gut there are both producers and users of vitamins. Vitamin B2 supplemented with the diet is phosphorylated to FMN and further metabolized to FAD (Powers, 2003) in the gut and then released in the blood and distributed throughout the body (Subramanian et al., 2016; Yoshii et al., 2019). On the other hand, bacterial vitamin B2 is synthesized from guanosine triphosphate (GTP) and D-ribulose 5-phosphate (García-Angulo, 2017), absorbed in gut, converted to FAD or FMN, and distributed as described above. In a metagenomic study on the human gut microbiota Magnúsdóttir et al. (2015) predicted that *Bacteroides fragilis* and *Prevotella copri* (Bacteroidetes) as well as *Clostridium difficile*, *Lactobacillus plantarum*, *L. fermentum* and *Ruminococcus lactaris* (Firmicutes) are vitamin B2 producers, while *Bifidobacterium* spp., and *Collinsella* spp. (Actinobacteria) lack a vitamin B2 pathway. Furthermore, Tastan (2017) showed that at phylum level, Bacteroidetes produce more riboflavin than Actinobacteria and Firmicutes. The biosynthesis of riboflavin can be achieved by eubacteria (e.g., *Bacillus subtilis* and *Escherichia coli*), yeasts (e.g., *Saccharomyces cerevisiae* and *Candida guilliermondii*) ascomycetes (e.g., *Ashbya gossypii*, *Eremothecium ashbyii*) (Bacher et al., 2001) and thermophilic *Geobacillus thermoglucosidasius* (Yang et al., 2021).

In a previous study we investigated the effects of supplementation of vitamin B2, in the form of riboflavin and riboflavin 5′-phosphate ester monosodium salt produced by *Bacillus subtilis*, on ileum and caeca microbiota of Ross 208. The impact on litter microbiota was tested as well (Biagi et al., 2020). Metabolomic analysis was performed on the caeca contents. Three groups of broilers were administered one diet each, containing 5 mg/kg (control group), +50 mg/kg (group B) and + 100 mg/kg (group C) of vitamin B. The two latter doses (i.e., x10 and x20 in comparison to the control diet) were selected to assess if in chickens there is a linear effect between vitamin B2 absorption and concentration as observed in humans (Steinert et al., 2016). The results of Biagi et al. showed that vitamin B2 significantly modulated the chicken microbiota, with the highest dose increasing the abundance of heath promoting bacteria groups. In the current study a group of caeca samples selected because of phylogenetic composition of the broiler’s microbiome in each group (i.e., no outliers, according to the 16S rRNA sequencing performed in Biagi et al., 2020) were submitted to shotgun metagenomic sequencing. The metagenomic and metabolomic features were analyzed by clustering and pathway enrichment to assess if these approaches can help to elucidate the specific biological impact of the vitamin B2 supplementation in the broiler diet on host caeca microbiome.

## 2. Materials and methods

### 2.1. Animals, experimental groups and sampling

The trial has been detailed in Biagi et al. (2020). Briefly, three experimental groups of 120 Ross 308 female each were housed in three separate rooms and fed with different diets (room A—control diet containing 5 mg/kg vitamin B2; room B—control diet +50 mg/kg vitamin B2; room C—control diet +100 mg/kg vitamin B2). Each experimental group was sampled at day 14 (T0), day 28 (T1), and day 42 (T2). During each sampling, a total of 40 birds/room were randomly selected and euthanized, following ethical guidelines described in Biagi et al. (2020) to minimize stress and pain. Caeca contents from the 120 birds were collected in duplicate for DNA extraction and NMR metabolome analyses performed as previously described (Biagi et al., 2020). At each sampling time, 21 DNA samples/room were selected and processed using shotgun metagenomic sequencing. They were selected based the 16S rRNA sequencing results detailed in Biagi et al. (2020) to avoid outliers. The DNA used for shotgun metagenomics was the same used in the previous study for the microbiota characterization.

### 2.2. Shotgun metagenomic sequencing

The 63 selected DNAs were fragmented and tagged with sequencing adapters using the Nextera XT DNA Library Preparation Kit (Illumina, San Diego, CA) resulting in libraries between 300 and 500 bp then processed by shotgun metagenomic sequencing in the NextSeq500 (Illumina) at 100 bp in paired-end mode. All shotgun metagenomic sequences tested as part of this study were deposited in MGRAST^1^ and are publicly available under the project named ProvaB2.^2^

### 2.3. Bioinformatics and statistics

Species-level characterization of shotgun metagenomic data was conducted as it follows. Shotgun reads were first filtered by quality and poultry sequences using the standard operating procedures of the HMP Consortium (Turnbaugh et al., 2007), with the GRCg6a genome as reference for the host genome. The obtained reads were taxonomically characterized at species level by MetaPhlAn2 (Truong et al., 2015). Metagenomes were functionally profiled using HUMAnN2 (Franzosa et al., 2018) to quantify abundance level of genes and pathways. Reads were aligned to sample-specific pangenomes, i.e., all gene families in any microorganism detected in a given sample, using Bowtie and the UniRef90, MinPath and KEGG databases (Wixon and Kell, 2000; Langmead et al., 2009; Ye and Doak, 2009; Suzek et al., 2015). Hits were counted per KEGG pathway and KO genes and normalized for length, alignment quality score and sequencing depth. Kruskall-Wallis and Bonferroni tests were used to evaluate differences between treatments and timepoints. Alpha and beta diversities were evaluated using the R package “vegan” (Oksanen et al., 2007). *p* values were corrected for multiple comparisons using the Benjamini–Hochberg method. False discovery rate (FDR) ≤ 0.05 was considered as statistically significant.

### 2.4. Data integration and clustering

Three types of features, coming from the different datasets, were integrated to characterize the effect of vitamin B2 in modulating caeca microbiome: (1) latent variables extracted from NMR spectra as described (Figure 6 in Biagi et al., 2020); (2) sample coordinates from the MDS axes of the Principal Coordinates Analysis (PCoA) performed on shotgun metagenomics data correlated with the species-level relative abundances (Supplementary Table S1); (3) abundance levels of functional pathways profiled from metagenomes (Supplementary Table S2). The pipeline for features integration, selection and clustering has been developed in Python version 3.8 using custom scripts and existing packages. Features were standard scaled before clustering. After scaling, features undergo a step of self-optimized features selection, using concurrent randomized decision trees (extra trees) to discriminate the subjects belonging to the different treated groups, for each time point. Features were selected when assigned a Gini importance strictly greater than the average of the Gini importance computed for all the features, weighted on the full ensemble of trees. Gini importance is defined and implemented in Scikit-learn as the *total decrease in node impurity*, weighted by the probability of reaching that node (which is approximated by the proportion of samples reaching that node), averaged over all trees of the ensemble as described by Breiman et al. (1984). Cluster maps were obtained with hierarchical bi-clustering with the Ward linking function, using Seaborn and Scikit-learn packages.

### 2.5. Network construction

**Figure 1:**
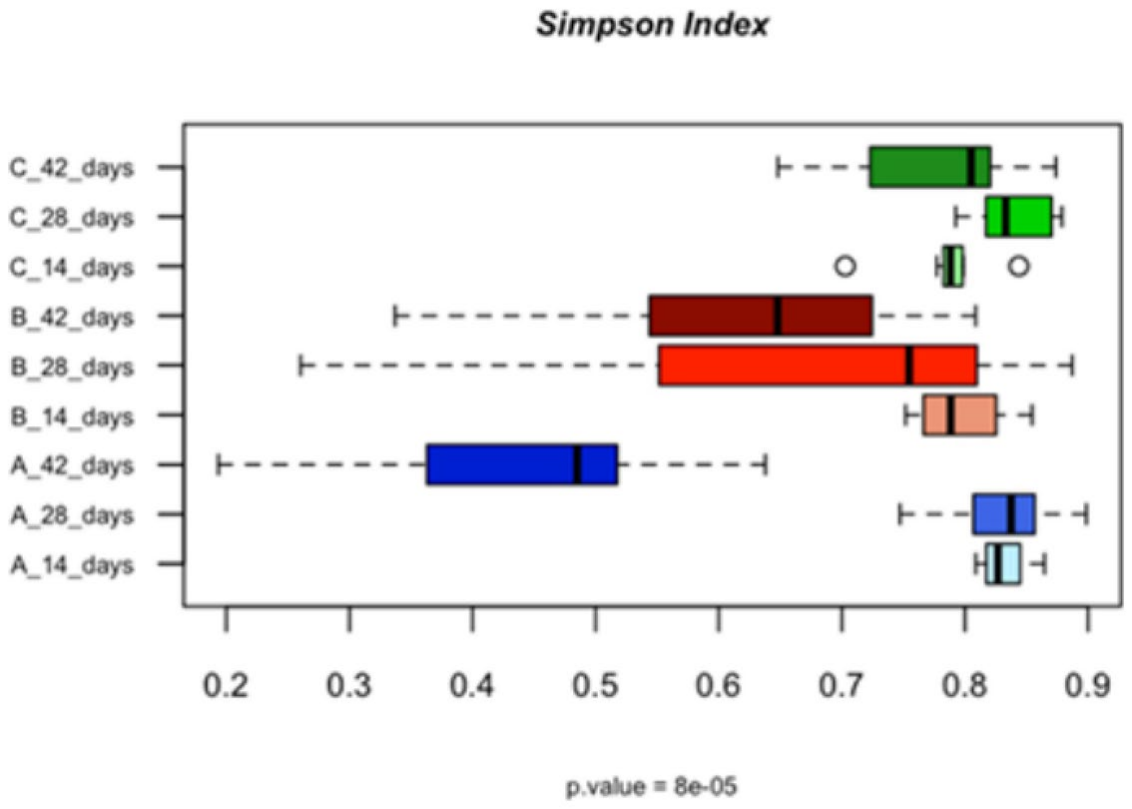
Bar plots showing the alpha diversity, calculated using the Simpson index, of the populations in the caeca of the birds fed with different levels of vitamin B2 (A—control diet containing 5 mg/kg vitamin B2; B—control diet +50 mg/kg vitamin B2; C—control diet +100 mg/kg vitamin B2) as quantified at the different sampling times (14, 28, and 42 days).

**Figure 2:**
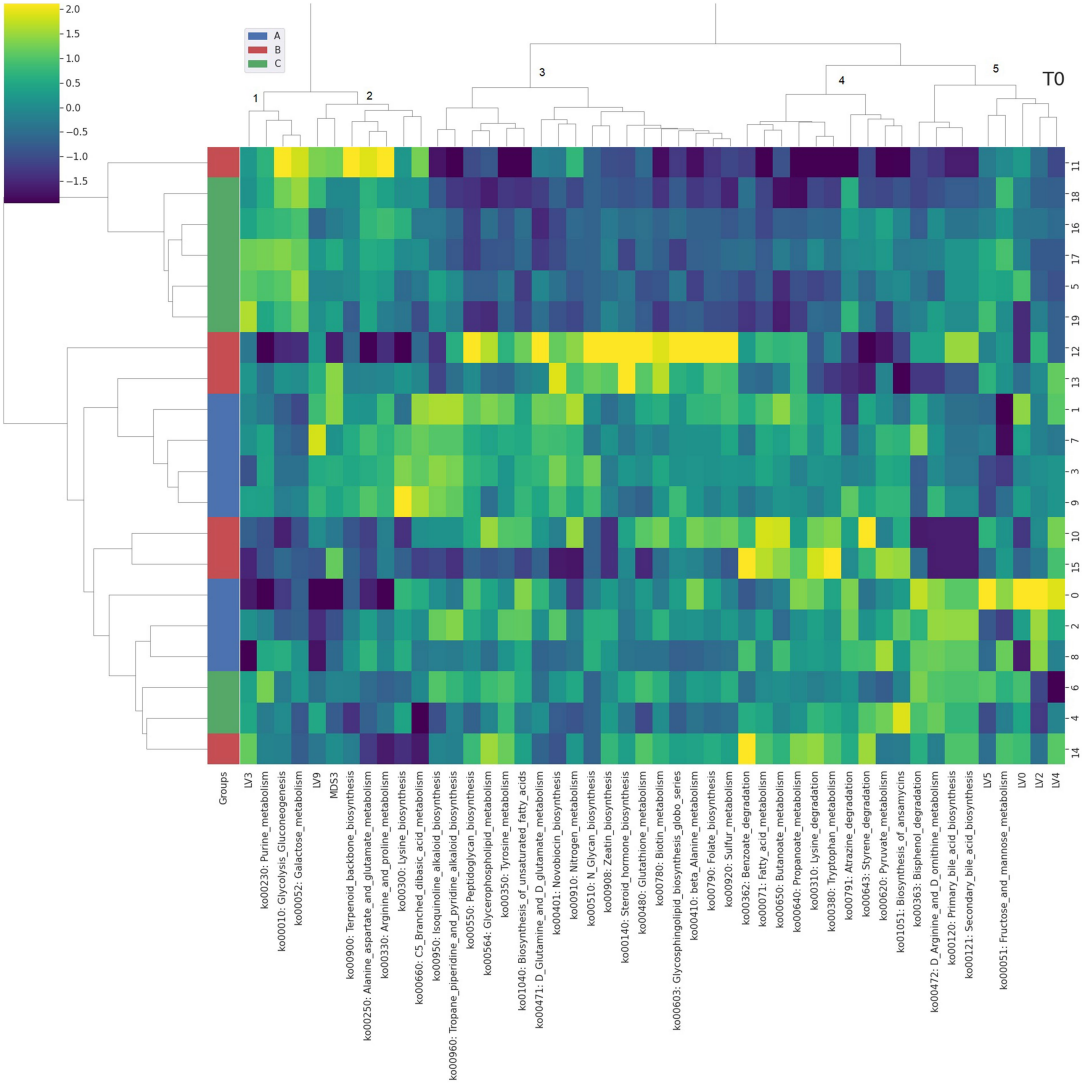
Integrated bi-clustering of features and subject at T0 (i.e., 14 days). Functional pathways are reported with their KEGG key entry. Identified clusters are indicated with numbers between 1 and 5. A in blue—control diet containing 5 mg/kg vitamin B2; B in red—control diet +50 mg/kg vitamin B2; C in green—control diet +100 mg/kg vitamin B2.

**Figure 3:**
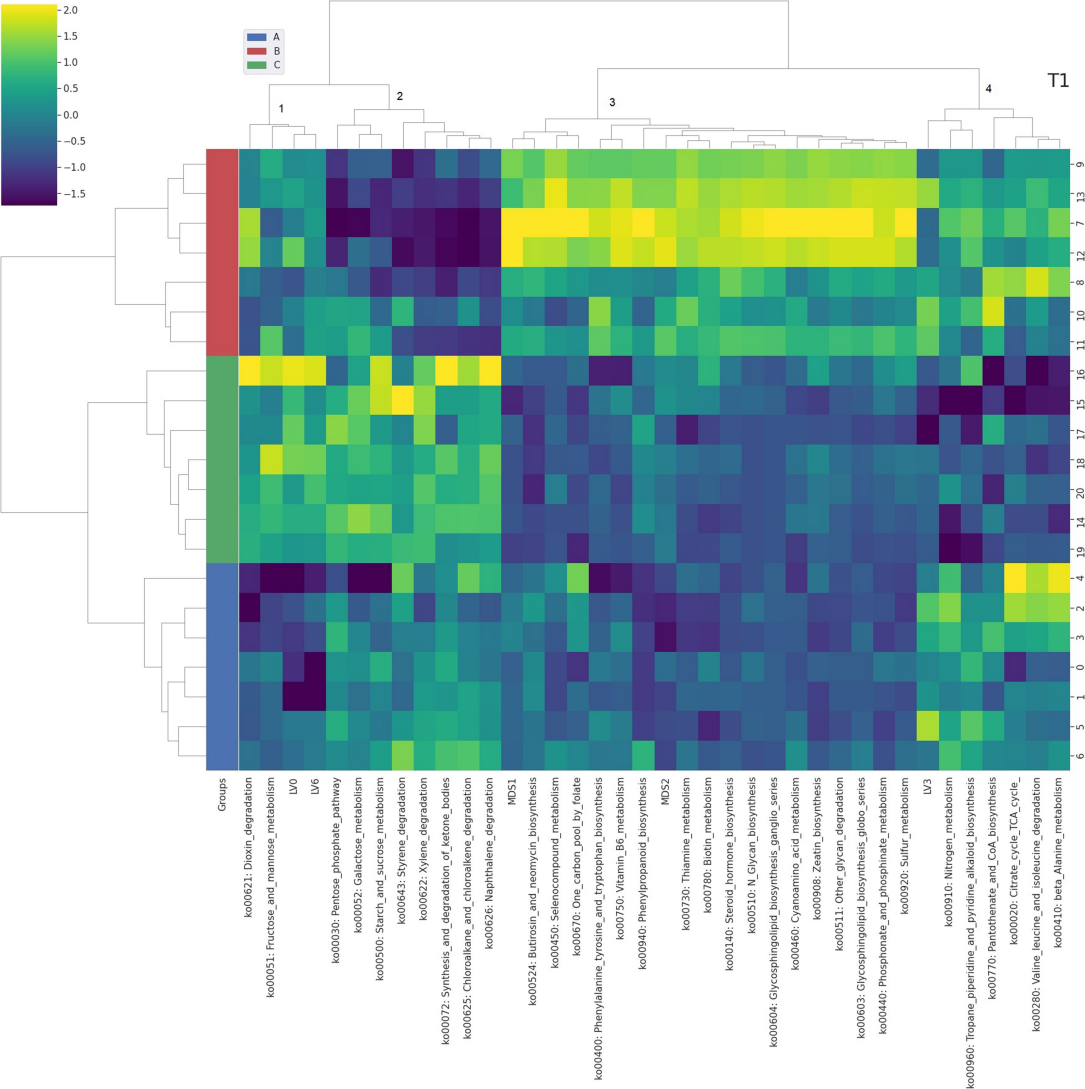
Integrated bi-clustering of features and subject at T1 (28 days). Functional pathways are reported with their KEGG key entry. Identified clusters are indicated with numbers between 1 and 5. A in blue—control diet containing 5 mg/kg vitamin B2; B in red—control diet +50 mg/kg vitamin B2; C in green— control diet +100 mg/kg vitamin B2.

Gene-Compound-Reaction-Enzyme networks were obtained using the MetScape plugin for the CytoScape 3.7.2 environment (Zhou et al., 2019). MetScape is capable of creating pathway-based networks by mapping lists of observed input genes and compounds mapped in KEGG, highlighting connected strands of metabolic ways through pathway-enrichment analysis. For each cluster map (one for each time point, Figures 1–3) a network was generated from each cluster of features containing at least an association between functional pathways and spectral latent variables (summarizing significative patterns of NMR-observed metabolites). This allowed to build a tool to investigate and describe biochemical mechanisms extrapolated from correlations amongst features resulting from the cluster maps. To build suitable inputs for MetScape, functional pathways were unpacked at gene level using a parsing custom script that automatically interrogates the KEGG API, translating KEGG orthologs into gene symbols and associating the corresponding ENTREZ ID when possible. Metabolite and compound names were automatically checked for suitable synonyms compatible with the MetScape framework.

## 3. Results

### 3.1. Impact of the different concentration of vitamin B2 on the taxonomic composition and functional genes identified in the broiler caeca over time

The supplementation of vitamin B2 started at day one in both treated groups. The taxonomic, functional and metabolomic features in the caeca of the birds showed an effect of the treatments since the first sampling, performed at day 14. The comparison between the taxonomic composition of the caeca between 14 and 28 days showed that *Bacteroides fragilis* was significantly more abundant in the caeca of birds fed +50 mg/kg vitamin B2 (*p* = 0.0001) (Supplementary Table S1), while in the caeca of birds fed +100 mg/kg vitamin B2 *Lactobacillus crispatus* bumped up to 12.40% of relative abundance since day 28, *Subdoligranum* spp. reached 20.19% at day 28 and *Ruminococcus torques* remained around 17% of relative abundance since day 28 until the end of the rearing cycle (Supplementary Table S1). The taxonomic groups supported in the control group were *Alistipes* spp., reaching 72% of relative abundance at the end of the trial.

At 42 days the supplementation of the highest concentration of vitamin B2 supported the relative abundance of *Lactobacillus crispatus* (*p* = 0.005), *Ruminococcus torques* (*p* = 0.002) and *Subdoligranum* spp. (*p* = 0.02) (Supplementary Table S1). *Bacteroides fragilis* was confirmed as most abundant in the caeca of the birds treated with +50 mg/kg of vitamin B2 (*p* = 0.0003) while *Alistipes* species were confirmed as bacterial species characterizing the caeca of birds belonging to the control group (*p* = 0.003). The microbial richness (alpha diversity) at the end of the rearing cycle was significantly higher in the treated groups in comparison to the control group (Figure 1).

The abundances of the entire pool of KEGG pathways statistically significantly different (*p* < 0.05, Kruskall-Wallis test) across our dataset are reported in Supplementary Table S2. The supplementation of +50 mg/kg of vitamin B2 at 28 days resulted in a higher abundance of genes involved in histidine metabolism, thiamine metabolism, peptidoglycan biosynthesis, nicotinate and nicotinamide metabolism. Moreover, at the end of the rearing cycle there was an increase of genes involved in thiamine metabolism and citrate cycle (Supplementary Table S2). The supplementation of +100 mg/kg vitamin B2 at 28 and 42 days resulted in a higher abundance of genes involved in purine metabolism, starch and sucrose metabolism, cystine and methionine metabolism, glycolysis and gluconeogenesis, pentose phosphate pathway and fructose and mannose metabolism. Moreover, at the end of the rearing cycle there was an increase of genes involved in pyrimidine metabolism. The control group at the end of the rearing cycle showed higher abundances of genes involved in glycerophospholipid metabolism, primary bile acids biosynthesis and peptidoglycan biosynthesis (Supplementary Table S2).

### 3.2. Mathematical relations between tested features as highlighted by the bi-clustering analysis

In order to find associations between metagenomic results and previously obtained metabolomic data (Biagi et al., 2020), we combined and integrated the followings in three different hierarchical clustering analyses (one for each timepoint): (1) latent variables extracted from the NMR spectra of metabolomic analysis (Supplementary Table S3); (2) PCoA ordination variables from species-level shotgun metagenomics (Supplementary Table S4); (3) abundance of functional genes profiled from metagenomes (Supplementary Table S2). Specifically, for each time point we identified features that clustered and varied together and that were characteristic of each group (if a sharp separation among groups was present).

**Figure 4:**
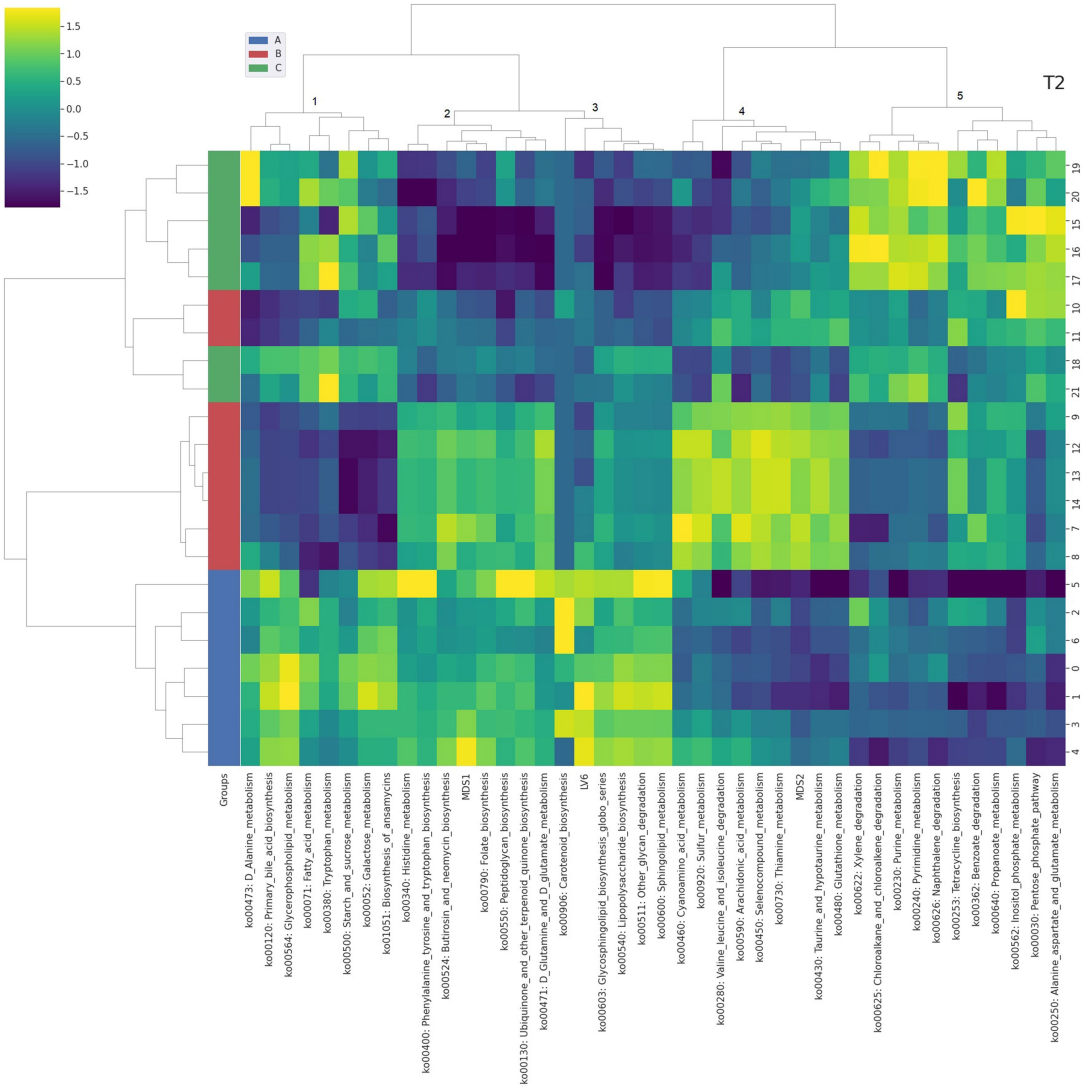
Integrated bi-clustering of features and subject at T2 (42 days). Functional pathways are reported with their KEGG key entry. Identified clusters are indicated with numbers between 1 and 5. A in blue—control diet containing 5 mg/kg vitamin B2; B in red—control diet +50 mg/kg vitamin B2; C in green—control diet +100 mg/kg vitamin B2.

At day 14 the representation of the taxonomic, functional and metabolomic features characterizing each tested group using clustering showed some degree of separation between control (group A, in blue) and each treated group (group B in red and group C in green) (Figure 2). On the contrary, at day 28 the separation between the three treatment groups (i.e., color bar on the left) was completed, showing a strong impact of the different concentrations of vitamin B2 (Figure 3). At day 42 the treatment group separation (color bar on the left) was still almost completed, indicating that the treatment effects were still detectable, although a complete separation between the two treated groups was lacking (Figure 4).

### 3.3. Biological relations between the tested features as highlighted by the network analysis and pathway enrichment

The Gene-Compound-Reaction-Enzyme network analysis was performed for the clusters of Figures 2–4 including both functional genes profiled from metagenomes and latent variables extracted from the NMR spectra (Supplementary Table S3). Each cluster containing at least one metabolomic latent variable (LV), in correlation with functional pathways, was selected for the pathway enrichment analysis. Metabolites contained in LVs were directly fed as inputs to the MetScape framework, while functional pathways were unpacked at KEGG genes level to be fed as inputs. The resulting enriched pathways are summarized in Table 1.

**Table 1:** Results of pathway enrichment analysis using features selected by the clustering analysis of metabolites contained in spectral LVs and functional pathways unpacked as KEGG Genes at the different sampling points.

Time	Cluster label	Metabolite	Resulting enriched pathways using KEGG genes from correlated functional pathways
T0	Cluster 1	Urocanate	Histidine metabolism
T0	Cluster 1	Xanthine	Purine metabolism
T0	Cluster 1	Hypoxanthine	Purine metabolism
T0	Cluster 1	Oxo-Iso Valerate	Valine, leucine and isoleucine degradation
T0	Cluster 2	Lactate	Glycolysis and gluconeogenesis
T0	Cluster 2	Nicotinate	Vitamin B3 (nicotinate and nicotinamide) metabolism
T0	Cluster 2	Xanthine	Purine metabolism
T0	Cluster 5	Butyrate/butanoate/butanoic acid	Butanoate metabolism
T0	Cluster 5	Formate	Biopterin metabolism
T0	Cluster 5	Formate	Urea cycle and metabolism of arginine, proline, glutamate, aspartate and asparagine
T0	Cluster 5	Formate	Vitamin B9 (folate) metabolism
T0	Cluster 5	Propanoate/propioni acid	Propanoate metabolism
T0	Cluster 5	Propanoate/propioni acid	Bile acid biosynthesis
T0	Cluster 5	L-Alanine	Glycine, serine, alanine and threonine metabolism
T0	Cluster 5	L-Alanine	Urea cycle and metabolism of arginine, proline, glutamate, aspartate and asparagine
T0	Cluster 5	Acetate	TCA cycle
T0	Cluster 5	Acetate	Glycolysis and gluconeogenesis
T0	Cluster 5	Acetate	Glycerophospholipid metabolism
T0	Cluster 5	Xanthine	Purine metabolism
T0	Cluster 5	Hypoxanthine	Purine metabolism
T0	Cluster 5	Nicotinate	Vitamin B3 (nicotinate and nicotinamide) metabolism
T0	Cluster 5	myo-Inositol	Phosphatidylinositol phosphate metabolism
T0	Cluster 5	Creatine Phosphate	Glycine, serine, alanine and threonine metabolism
T1	Cluster 1	L-Tyrosine	Biopterin metabolism
T1	Cluster 1	L-Tyrosine	Tyrosine metabolism
T1	Cluster 1	Acetate	TCA cycle
T1	Cluster 1	Acetate	Glycolysis and gluconeogenesis
T1	Cluster 1	Acetate	Glycerophospholipid metabolism
T1	Cluster 1	Myo-Inositol	Phosphatidylinositol phosphate metabolism
T1	Cluster 1	Lactate	Glycolysis and gluconeogenesis
T1	Cluster 1	Nicotinate	Vitamin B3 (nicotinate and nicotinamide) metabolism
T1	Cluster 4	Urocanate	Histidine metabolism
T1	Cluster 4	Oxo-Iso Valerate	Valine, leucine and isoleucine degradation
T1	Cluster 4	Xanthine	Purine metabolism
T2	Cluster 3	L-Tyrosine	Tyrosine metabolism
T2	Cluster 3	L-Tyrosine	Biopterin metabolism
T2	Cluster 3	Lactate	Glycolysis and gluconeogenesis
T2	Cluster 3	Nicotinate	Vitamin B3 (nicotinate and nicotinamide) metabolism

In the following section, the in-depth comprehensive results of features integration framework are discussed for three metabolic pathways selected for their biological relevance, the significance of differences detected at the various level (functional pathways, NMR signals of observed metabolites) and the overlap of information in the resulting pathway enriched networks.

**Figure 5:**
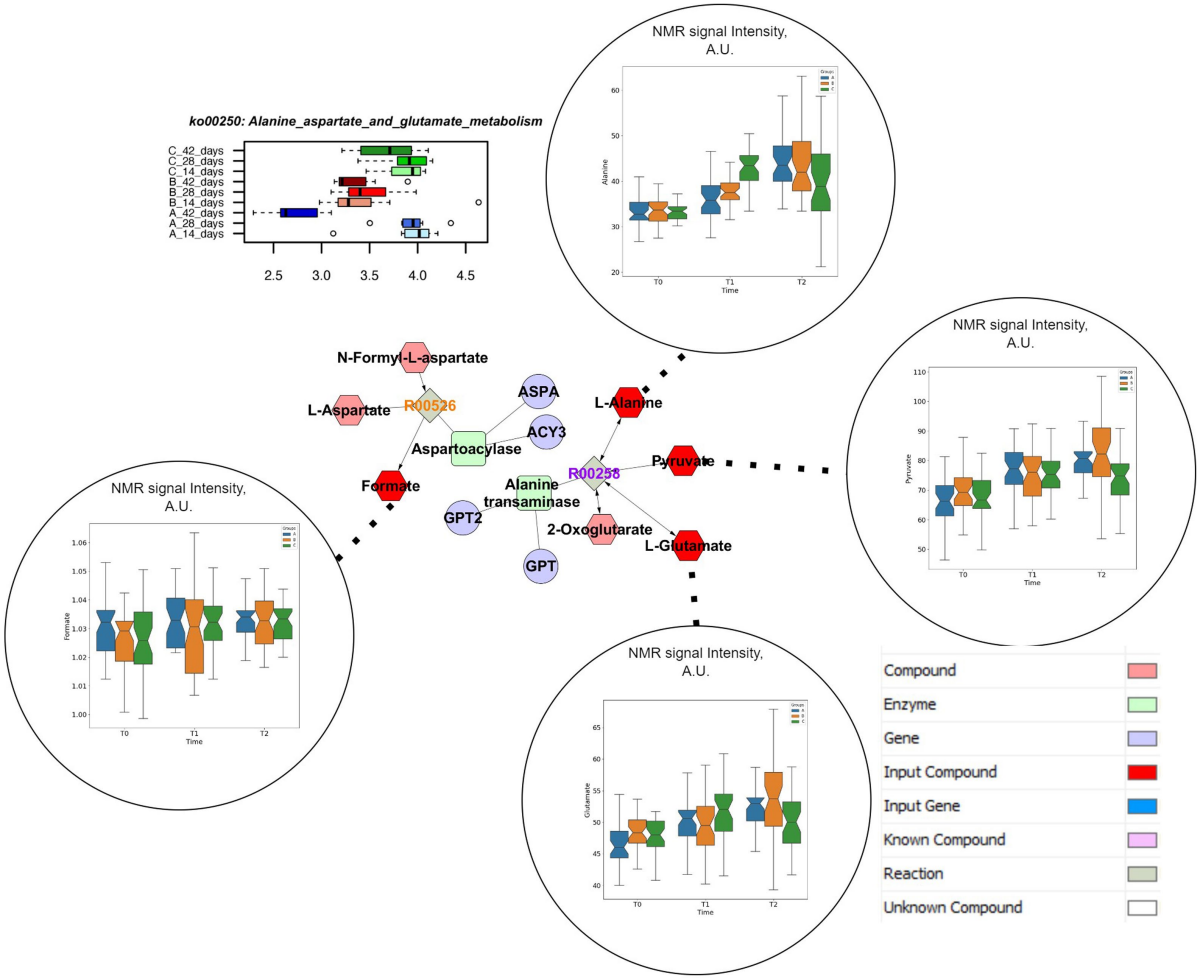
Comprehensive results for Alanine, Aspartate and Glutamate metabolism. This metabolic pathway is significantly impacted in different treatment groups at functional pathway level (upper left). Clustering and pathway enrichment analysis adds significant information of NMR observed metabolites (dark red) that are linked in the resulting pathway enriched network to the same metabolic way, as a subunit of the urea cycle.

Both concentrations of vitamin B2 supported the abundance of genes coding for alanine, aspartate and glutamate metabolism in the caeca of the chickens up to the end of the rearing cycle (Supplementary Table S2). The pathway enrichment analysis showed that the specific reactions enhanced by the supplementations of vitamin B2 are the N-Formyl-L-aspartate amidohydrolase (R00526), producing Aspartate and Formate, and L-Alanine:2-oxoglutarate aminotransferase (R00258) supporting the conversion of L-Alanine and 2-Oxoglutarate in Pyruvate and L-Glutamate (Figure 5). The analysis of spectral signals associated to Alanine and Glutamate resulted in significantly higher levels of these two metabolites in the caeca at 28 days for the group treated with highest doses of vitamin B2 (group C). At the end of the cycle, Glutamate and Pyruvate were significantly lower in samples from group C.

**Figure 6:**
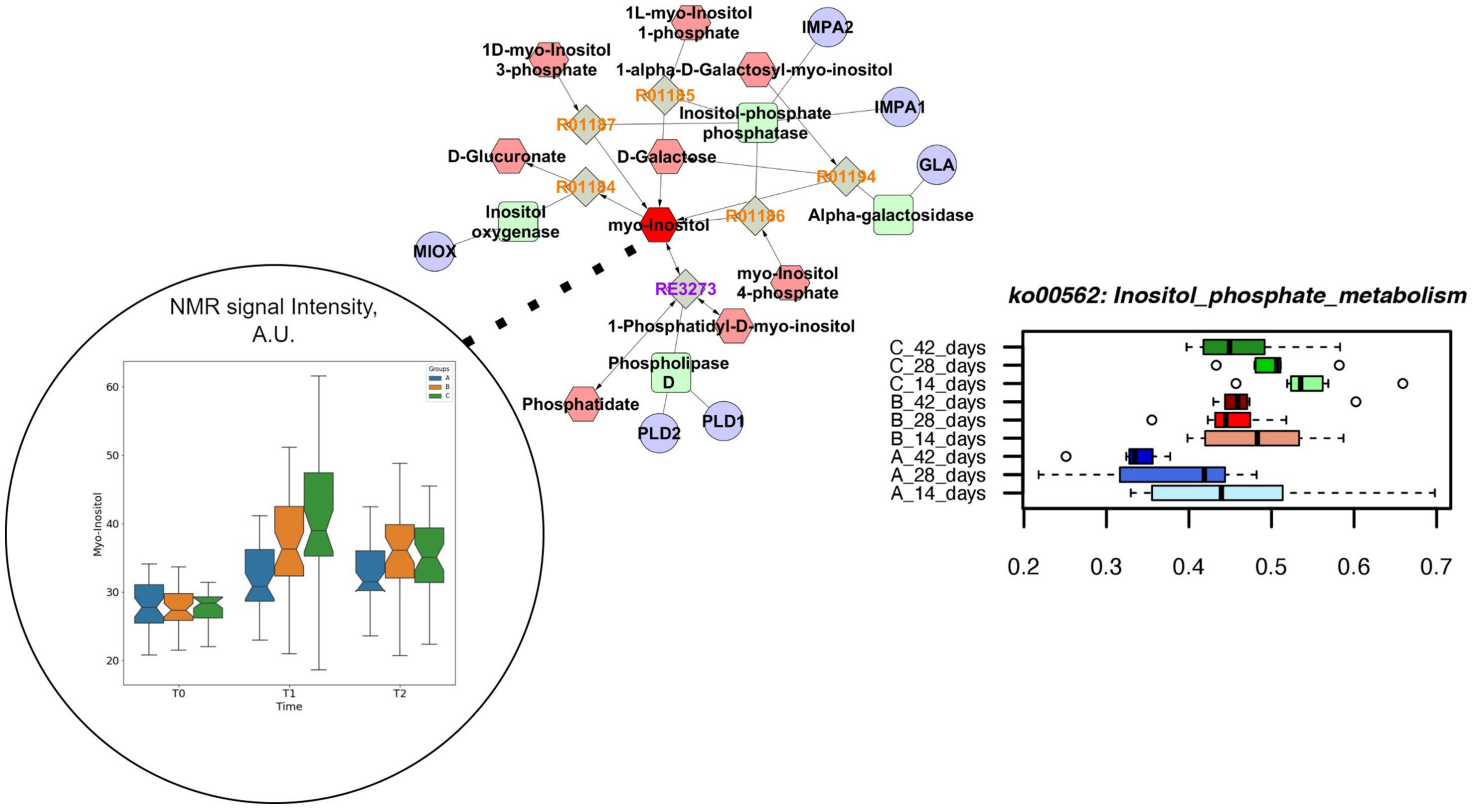
Comprehensive results for inositol phosphate metabolism. This metabolic path is significantly impacted in different treatment group at functional pathway level (lower right). Clustering and pathway enrichment analysis adds significant information of NMR observed myo-inositol (dark red), that is linked in the resulting pathway enriched network to the same metabolic way.

**Figure 7:**
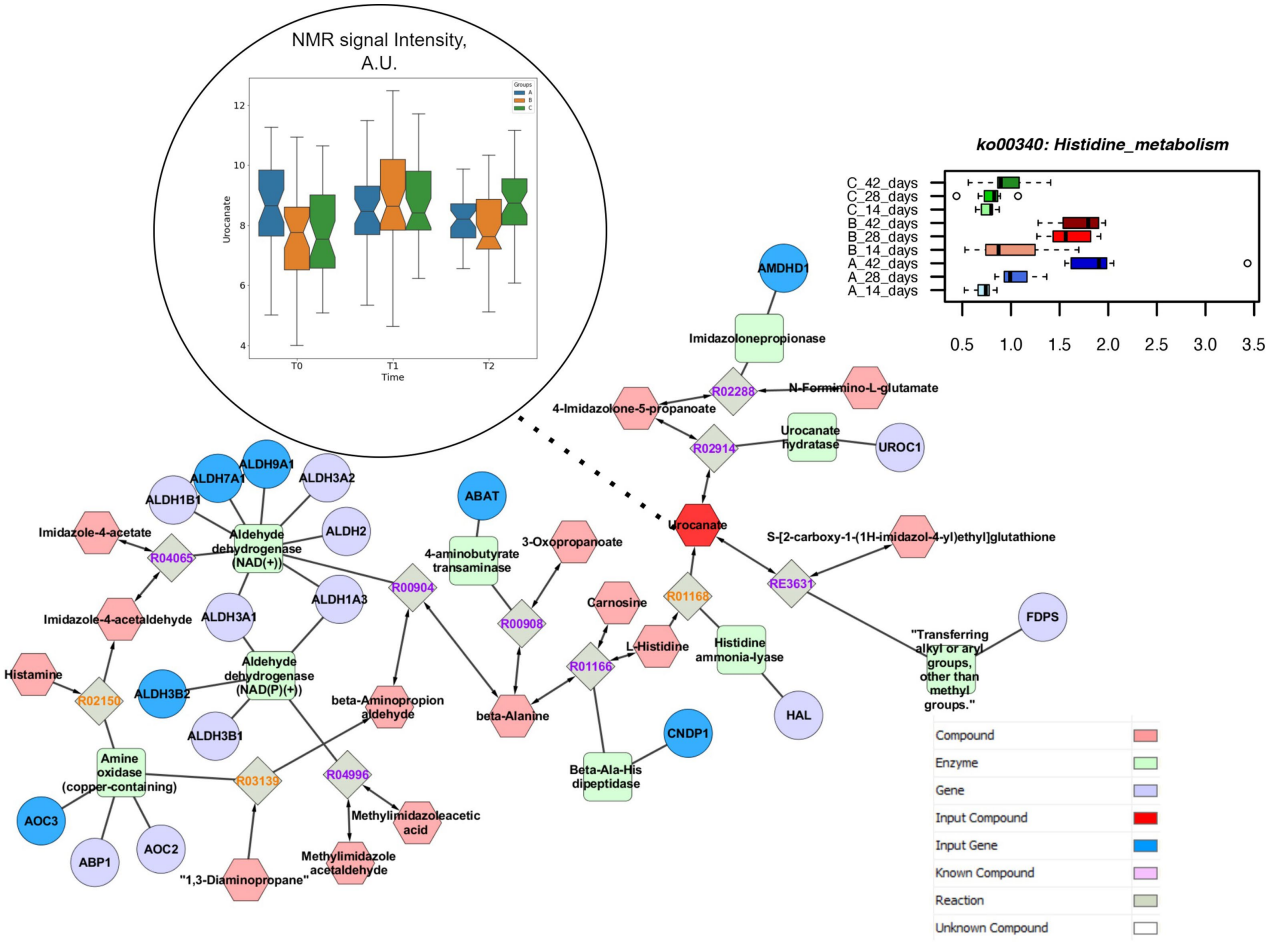
Comprehensive results for histidine metabolism. This metabolic path is significantly impacted in different treatment group at functional pathway level (upper right). Clustering and pathway enrichment analysis adds significant information of NMR observed urocanate (dark red), that is linked in the resulting pathway enriched network, with several input genes (dark blue) to the same metabolic way.

Both concentrations of vitamin B2 increased the genes coding for inositol phosphate metabolism up to the end of the rearing cycle, in comparison to the control group (Figure 6). The pathway enrichment analysis showed that the specific reactions enhanced by the supplementations of vitamin B2 and resulting in the higher production of myo-inositol were the 1D-myo-inositol 1-phosphate phosphohydrolase (R01185), converting Inositol 1-phosphate and water in myo-Inositol and Orthophosphate as well as 1D-myo-inositol 4-phosphate phosphohydrolase (R01186), converting myo-Inositol 4-phosphate and water in myo-Inositol and Orthophosphate. The higher concentration of vitamin B2 significantly decreased the relative abundance of genes coding for histidine metabolism (Supplementary Table S2). The pathways enrichment showed that the reaction linking the histidine to urocanate was the L-histidine ammonia-lyase (R01168), converting L-histidine in urocanate and ammonia. However, urocanate is also produced by the reaction 4,5-Dihydro-4-oxo-5-imidazolepropanoate hydro-lyase (R02914), converting the 4-Imidazolone-5-propanoate in urocanate and water. The different pathways resulting in urocanate production explained why in the group +100 mg/kg B2 the level of urocanate did not significantly decrease in the caeca in comparison to both the +50 mg/kg B2 and the control as quantified by metabolomics (Figure 7).

## 4. Discussion

Understanding the effect of chicken nutritional treatments on the gut microbiome and metabolome is expected to provide new strategies to rear healthy animals, reducing the need of antimicrobial therapeutic treatments during their life cycle, thus resulting in more sustainable poultry productions. In this study shotgun metagenomics and metabolomics were applied to investigate the impact of changes in vitamin B2 concentration in the broiler diet on caeca microbiome. Vitamin B2 can be both produced and used by microbiome members. In the human gut it was observed that the majority of microbial synthesized B vitamins is used by non-producing vitamin B microbes, thus reducing their availability for the host (Magnúsdóttir et al., 2015). In the chicken gut this cooperation between microbiome members is unknown, as well as the impact on the supplementation of exogenous vitamin B at different concentrations. The chicken gut holds a complex community of microorganisms, changing during the animal life cycle, following dynamics affected by dietary treatments. Shotgun metagenomics can be used to map these changes in combination with metabolomics. These omics methods return complex results which are difficult to relate and integrate one to the other to clarify meaningful biological effects of nutritional interventions.

The approach followed in this paper explains how to perform multi-omic features integration, aiming at the description of the biochemical mechanisms, rather than simple statistical correlations, among different omic measures. The first part of the framework was an automated pipeline for features selection, based on randomized concurrent decision trees clustering (1) metabolomic features, (2) microbial species, and (3) functional pathways. The features selection was performed in order to obtain a clustering of the samples with minimal sets of meaningful inter-omic correlated features that can be explored to detect the effect of vitamin B2 supplementation in biochemical pathways. To do this, the second part of the framework relied upon unpacking functional pathways at gene level using the KEGG API, along with compounds identified in NMR spectral components. These inputs were then fed to a pathway enrichment-based network model built with the MetScape tool. The result was a series of networks of connected pathways underlying which biological functions were affected by the tested treatments.

In the caeca of birds belonging to the control group fed a baseline dose of vitamin B2, corresponding to 5 mg/kg, *Alistipes* spp. was the signature species. This microorganism plays a role in energy metabolism as well as amino acid, nucleotide and short chain fatty acid (SCFA) utilization and has been identified as prevalent in conventional poultry farms by other authors (McKenna et al., 2020). The heat maps and Supplementary Table S2 showed that the functional genes involved in glycerophospholipid metabolism, primary bile acid biosynthesis and peptidoglycan biosynthesis were significantly more abundant in the control group at the end of the rearing cycle. The genes involved in peptidoglycan biosynthesis are involved in the production of the DAP-type peptidoglycan often found in the peptide linkages of NAM-NAG chains that make up the cell wall of Gram-negative bacteria (Brooks, 2004). This result can explain the significative higher abundance of *Alistipes* in the control group in comparison to the treated groups at the end of the rearing cycle (72.38 vs. 5.34 and 25.87%). The primary bile acids are important for absorption of dietary fat and fat-soluble vitamins from entero-hepatic circulations (Uebanso et al., 2020) and according to our results they are not supported by additional concentrations of vitamin B2.

The signature species identified in the caeca of birds fed +50 mg/kg vitamin B2 was *Bacteroides fragilis*. The abundance of this species in presence of vitamins is well described in the literature (Lauridsen et al., 2021; Hossain et al., 2022). This microorganism is a vitamin B producer and has a complete riboflavin operon (Hossain et al., 2022). The overall increase of genes involved in the thiamine metabolism observed in the caeca of the animals belonging to the +50 mg/kg B2 group can be explained by the enhancement of pyridoxal (vitamin B6) supported by flavin adenine dinucleotide (FAD) derived from vitamin B2 metabolism because the thiamine metabolism is related to purine metabolism and pyridoxal (vitamin B6) metabolism.

The signature bacteria of group C at the end of the rearing cycle were four species of the genus *Lactobacillus*, *Ruminococcus torques* and both *Subdoligranum variabile* and other *Subdoligranum* species. The impact of vitamin B2 on strictly anaerobic bacteria has been observed by other authors (Luo et al., 2013) and can be explained considering that riboflavin as redox mediator can reduce the oxidative stress. The higher abundance of *Lactobacillus* in the caeca of broilers fed vitamins has been also described by Luo et al. (2013). These authors suggested that the presence of dietary vitamins decrease the ratio of pathogenic bacteria and increase the diversity of bacteria in the caeca, as we also demonstrate in Figure 1. Overall, the increase of abundance of the functional genes involved in alanine, aspartate and glutamate metabolism promotes the increase of TCA cycle, which is justified by the high riboflavin availability in groups +50 and +100 mg/kg B2.

The higher abundance of genes involved in the purine metabolism in the caeca of the birds fed +100 mg/kg B2 can be explained considering that all the genes linked to purine pathway promote the biosynthesis of riboflavin and other nucleotides representing essential metabolites for nucleic acid synthesis, energy supply and biosynthesis of several amino acids (Smith et al., 1994). The biosynthetic relationship between purines and vitamins has been described (Dmytruk et al., 2020). This relationship explains because in the caeca of the birds fed +100 mg/kg B2 there was a higher concentration of hypoxanthine and xanthine, which are purine intermediates from central carbon metabolism (Peifer et al., 2012), as well as trimethylamine and oxoisovalerate.

The application of the pathway enrichment modelling highlighted that the supplementation of vitamin B2 at the concentration of +50 mg/kg supported the production of myo-inositol, aspartate, glutamate, formate and pyruvate. In the past myo-inositol was considered a member of the vitamin B group but this hypothesis was discharged because chickens can biosynthesize myo-inositol (Regidor and Schindler, 2016). There is a lack of information on the impact of the myo-inositol on poultry performances and metabolism but it seems to act as growth promoter (Cowieson and Zhai, 2021). A review on myo-inositol metabolism and its potential implications for poultry nutrition demonstrated that in broilers it enhances mineral adsorption, bone mineralization, skeletal muscle glucose uptake and breast muscle development (Gonzalez-Uarquin et al., 2020). However, the relationship between vitamin B2 supplementation and myo-inositol production was highlighted in our study for the first time. Concerning the other metabolites enhanced by presence of vitamin B2, aspartate and glutamate are essential amino acids for the protein production and act as excitatory neurotransmitters (Tomonaga and Furuse, 2020) while pyruvate is a key metabolite of glycolysis and is involved in the alanine, aspartate and glutamate metabolism, lysine biosynthesis, as well as glycine, serine and threonine metabolism (Zhang et al., 2020).

## 5. Conclusion

All in all, the results of this study showed that the use of multi-omic features clustering to select the inputs (i.e., genes from functional pathways and observed metabolites) for the pathway enrichment analysis allowed to identify the specific biochemical reactions impacting the concentration of myo-inositol, formic acid, amino acids and pyruvate in the caeca of the birds fed the highest doses of vitamin B2. This approach, based on data integration, can be exploited to interpret complex datasets from studies where multi-omic technologies are applied to discover the biological mechanisms of nutritional interventions.

## Data availability statement

The datasets presented in this study can be found in online repositories. The names of the repository/repositories and accession number(s) can be found at this link: https://www.mg-rast.org/linkin.cgi?project=mgp89032.

## Ethics statement

The animal study was approved by Ethical Committee of the University of Bologna (Protocol ID 881/2019). The study was conducted in accordance with the local legislation and institutional requirements.

## Author contributions

CM: Writing – original draft, Data curation, Formal analysis, Writing – review & editing. SR: Data curation, Formal analysis, Writing – original draft, Writing – review & editing. GP: Writing – review & editing, Investigation. AL: Investigation, Writing – review & editing. GL: Conceptualization, Writing – review & editing. EB: Investigation, Writing – review & editing. MC: Investigation, Writing – review & editing. GM: Conceptualization, Writing – review & editing. PB: Conceptualization, Writing – review & editing. FC: Conceptualization, Writing – review & editing. AC: Investigation, Supervision, Writing – original draft, Writing – review & editing.

## Footnotes

^1^ http://metagenomics.anl.gov/

^2^ https://www.mg-rast.org/linkin.cgi?project=mgp89032
