# Supplementary material for: Application of multi-omic features clustering and pathway enrichment to clarify the impact of vitamin B2 supplementation on broiler caeca microbiome
Source: Front Microbiol. 2023 Sep 28;14:1264361. doi: 10.3389/fmicb.2023.1264361 (PMC10568133; doi:10.3389/fmicb.2023.1264361)
Supplement: Supplementary file 1 [file Table_1.DOCX]

Table 1S. Relative abundance of bacteria species detected as significantly different between tested groups (Group A: control group; Group B: + 50 mg/kg vitamin B2; Group C: + 100 mg/kg vitamin B2) and sampling times (T0: day 14; T1: day 28; T2: day 42).

|  | Control  14 days | | Control  28 days | | Control  42 days | | +50 mg/kg B2  14 days | | +50 mg/kg B2  28 days | | +50 mg/kg B2  42 days | | +100 mg/kg B2  14 days | | +100 mg/kg B2 28 days | | +100 mg/kg B2 42 days | |
| --- | --- | --- | --- | --- | --- | --- | --- | --- | --- | --- | --- | --- | --- | --- | --- | --- | --- | --- |
| Bacterial species | Mean | SD | Mean | SD | Mean | SD | Mean | SD | Mean | SD | Mean | SD | Mean | SD | Mean | SD | Mean | SD |
| *Propionibacterium_acnes* | 0.549 | 0.620 | 0.557 | 0.378 | 0.183 | 0.300 | 0.237 | 0.393 | 0.204 | 0.349 | 0.040 | 0.070 | 0.019 | 0.051 | 0.009 | 0.025 | 0.000 | 0.000 |
| *Bacteroides_fragilis* | 0.000 | 0.000 | 0.000 | 0.000 | 6.715 | 10.401 | 14.675 | 20.255 | 48.077 | 25.591 | 54.308 | 20.877 | 0.000 | 0.000 | 0.000 | 0.000 | 0.000 | 0.000 |
| *Alistipes_spp* | 0.000 | 0.001 | 25.095 | 8.627 | 72.384 | 11.535 | 0.000 | 0.000 | 7.579 | 6.632 | 5.340 | 2.347 | 0.000 | 0.000 | 7.635 | 5.942 | 25.872 | 21.856 |
| *Lactobacillus_crispatus* | 0.632 | 0.956 | 1.953 | 2.167 | 1.322 | 1.654 | 2.437 | 3.555 | 3.260 | 4.028 | 3.242 | 2.475 | 1.258 | 0.807 | 12.398 | 10.259 | 12.401 | 13.020 |
| *Lactobacillus_reuteri* | 0.013 | 0.034 | 0.013 | 0.034 | 0.110 | 0.120 | 0.008 | 0.021 | 0.037 | 0.096 | 0.148 | 0.232 | 0.022 | 0.058 | 0.391 | 0.757 | 1.029 | 1.133 |
| *Pseudoflavonifractor_capillosus* | 3.360 | 1.132 | 2.412 | 0.720 | 0.296 | 0.144 | 2.813 | 2.105 | 0.547 | 0.341 | 0.419 | 0.250 | 4.039 | 1.649 | 1.622 | 0.931 | 0.955 | 0.553 |
| *Ruminococcus_torques* | 21.239 | 12.505 | 12.307 | 3.853 | 2.648 | 2.257 | 15.531 | 11.574 | 4.951 | 2.763 | 8.918 | 4.931 | 34.469 | 9.525 | 16.856 | 7.764 | 17.494 | 12.948 |
| *Lachnospiraceae_bacterium_1_1_57FAA* | 0.660 | 0.431 | 0.515 | 0.581 | 0.065 | 0.037 | 0.299 | 0.280 | 0.188 | 0.260 | 0.140 | 0.092 | 0.431 | 0.210 | 0.915 | 0.677 | 0.283 | 0.223 |
| *Lachnospiraceae_bacterium_8_1_57FAA* | 0.366 | 0.348 | 0.192 | 0.237 | 0.017 | 0.019 | 0.000 | 0.000 | 0.085 | 0.172 | 0.051 | 0.081 | 0.468 | 0.563 | 0.482 | 0.585 | 0.647 | 0.886 |
| *Oscillibacter_spp* | 10.002 | 7.441 | 9.230 | 7.796 | 2.052 | 1.425 | 10.350 | 8.860 | 4.353 | 3.922 | 2.194 | 2.379 | 8.634 | 3.458 | 6.986 | 3.848 | 4.185 | 2.130 |
| *Anaerotruncus_colihominis* | 15.387 | 10.193 | 10.996 | 13.783 | 0.492 | 0.620 | 16.699 | 14.366 | 6.613 | 5.847 | 3.247 | 4.166 | 18.190 | 11.384 | 7.828 | 10.703 | 2.649 | 3.604 |
| *Anaerotruncus_spp* | 0.292 | 0.730 | 1.260 | 1.377 | 0.182 | 0.183 | 2.234 | 2.478 | 0.840 | 0.824 | 0.106 | 0.141 | 0.000 | 0.000 | 0.247 | 0.477 | 0.088 | 0.134 |
| *Faecalibacterium_prausnitzii* | 3.652 | 2.200 | 5.134 | 2.838 | 1.767 | 1.462 | 2.439 | 4.801 | 1.873 | 1.632 | 1.023 | 1.325 | 2.272 | 1.744 | 1.822 | 0.782 | 1.168 | 1.099 |
| *Subdoligranulum_spp* | 11.635 | 5.083 | 16.300 | 6.369 | 4.051 | 3.197 | 12.586 | 8.628 | 13.476 | 7.415 | 11.983 | 12.467 | 13.718 | 5.502 | 20.189 | 8.967 | 15.272 | 5.674 |
| *Erysipelotrichaceae_bacterium_21_3* | 0.417 | 0.224 | 0.632 | 0.744 | 0.081 | 0.074 | 0.259 | 0.239 | 0.148 | 0.177 | 0.108 | 0.072 | 0.591 | 0.523 | 0.495 | 0.305 | 0.520 | 0.333 |
| *Erysipelotrichaceae_bacterium_2_2_44A* | 0.208 | 0.218 | 0.181 | 0.191 | 0.053 | 0.066 | 0.015 | 0.019 | 0.092 | 0.081 | 0.165 | 0.108 | 0.233 | 0.112 | 0.397 | 0.383 | 0.535 | 0.347 |
| *Escherichia_coli* | 7.868 | 6.555 | 2.374 | 3.022 | 1.706 | 3.340 | 8.573 | 6.487 | 1.249 | 1.027 | 1.627 | 1.806 | 3.960 | 3.856 | 4.827 | 4.735 | 3.631 | 3.325 |

Table 2S. KEGG pathways (mean ± standard deviation) showing statistically significant differences between treated (+50 and 100 mg/kg B2) and control (5 mg/kg B2) groups. Kw= Kruskal-Wallis test; B=Bonferroni test.

| Pathways | Control  14 days | | +50 mg/kg B2  14 days | | +100 mg/kg B2 14 days | | Control  28 days | | +50 mg/kg B2 28 days | | +100 mg/kg B2 28 days | | Control  42 days | | +50 mg/kg B2 42 days | | +100 mg/kg B2 42 days | | Kw | B |
| --- | --- | --- | --- | --- | --- | --- | --- | --- | --- | --- | --- | --- | --- | --- | --- | --- | --- | --- | --- | --- |
|  | Mean | SD | Mean | SD | Mean | SD | Mean | SD | Mean | SD | Mean | SD | Mean | SD | Mean | SD | Mean | SD |  |  |
| ko00791: Atrazine_degradation | 0.013 | 0.007 | 0.006 | 0.005 | 0.011 | 0.002 | 0.535 | 0.327 | 0.242 | 0.280 | 0.205 | 0.147 | 0.395 | 0.091 | 0.148 | 0.059 | 0.384 | 0.204 | 0.000 | 0.000 |
| ko00230: Purine_metabolism | 6.632 | 0.489 | 6.341 | 0.440 | 7.035 | 0.250 | 7.634 | 0.510 | 6.567 | 0.675 | 7.854 | 0.325 | 5.738 | 0.516 | 6.249 | 0.394 | 7.869 | 0.410 | 0.000 | 0.000 |
| ko00400: Phenylalanine_tyrosine_and_tryptophan_biosynthesis | 1.440 | 0.126 | 1.527 | 0.351 | 1.279 | 0.079 | 1.096 | 0.164 | 1.689 | 0.191 | 1.051 | 0.106 | 1.929 | 0.520 | 1.737 | 0.141 | 1.035 | 0.185 | 0.000 | 0.000 |
| ko00140: Steroid_hormone_biosynthesis | 0.006 | 0.003 | 0.047 | 0.050 | 0.005 | 0.006 | 0.001 | 0.001 | 0.087 | 0.033 | 0.003 | 0.003 | 0.222 | 0.041 | 0.107 | 0.033 | 0.043 | 0.055 | 0.000 | 0.000 |
| ko00401: Novobiocin_biosynthesis | 0.195 | 0.037 | 0.177 | 0.070 | 0.128 | 0.020 | 0.148 | 0.031 | 0.224 | 0.038 | 0.094 | 0.028 | 0.374 | 0.173 | 0.239 | 0.036 | 0.117 | 0.037 | 0.000 | 0.000 |
| ko00511: Other_glycan_degradation | 0.046 | 0.015 | 0.252 | 0.300 | 0.043 | 0.021 | 0.026 | 0.016 | 0.578 | 0.307 | 0.051 | 0.026 | 2.121 | 0.557 | 0.730 | 0.282 | 0.430 | 0.481 | 0.000 | 0.000 |
| ko00340: Histidine_metabolism | 0.713 | 0.113 | 1.011 | 0.409 | 0.760 | 0.088 | 1.055 | 0.187 | 1.608 | 0.250 | 0.788 | 0.196 | 2.014 | 0.654 | 1.703 | 0.268 | 0.966 | 0.271 | 0.000 | 0.000 |
| ko00603: Glycosphingolipid_biosynthesis_globo_series | 0.073 | 0.025 | 0.190 | 0.209 | 0.035 | 0.014 | 0.049 | 0.022 | 0.388 | 0.217 | 0.036 | 0.013 | 0.877 | 0.192 | 0.499 | 0.183 | 0.187 | 0.199 | 0.000 | 0.000 |
| ko00531: Glycosaminoglycan_degradation | 0.006 | 0.002 | 0.084 | 0.122 | 0.003 | 0.003 | 0.005 | 0.004 | 0.215 | 0.126 | 0.004 | 0.004 | 0.708 | 0.168 | 0.271 | 0.106 | 0.131 | 0.171 | 0.000 | 0.000 |
| ko00785: Lipoic_acid_metabolism | 0.027 | 0.009 | 0.126 | 0.127 | 0.026 | 0.022 | 0.136 | 0.071 | 0.290 | 0.097 | 0.064 | 0.026 | 0.272 | 0.073 | 0.339 | 0.100 | 0.124 | 0.056 | 0.000 | 0.000 |
| ko00950: Isoquinoline_alkaloid_biosynthesis | 0.129 | 0.018 | 0.080 | 0.022 | 0.102 | 0.017 | 0.089 | 0.019 | 0.039 | 0.026 | 0.065 | 0.022 | 0.036 | 0.022 | 0.029 | 0.021 | 0.054 | 0.021 | 0.000 | 0.000 |
| ko00790: Folate_biosynthesis | 0.178 | 0.057 | 0.402 | 0.346 | 0.092 | 0.049 | 0.230 | 0.075 | 0.708 | 0.306 | 0.167 | 0.082 | 0.994 | 0.175 | 0.855 | 0.282 | 0.358 | 0.198 | 0.000 | 0.000 |
| ko00510: N_Glycan_biosynthesis | 0.007 | 0.006 | 0.027 | 0.043 | 0.000 | 0.000 | 0.001 | 0.002 | 0.077 | 0.040 | 0.000 | 0.001 | 0.082 | 0.027 | 0.094 | 0.034 | 0.013 | 0.016 | 0.000 | 0.000 |
| ko00604: Glycosphingolipid_biosynthesis_ganglio_series | 0.000 | 0.000 | 0.078 | 0.123 | 0.000 | 0.000 | 0.001 | 0.000 | 0.214 | 0.126 | 0.000 | 0.001 | 0.706 | 0.170 | 0.270 | 0.108 | 0.128 | 0.172 | 0.000 | 0.000 |
| ko00240: Pyrimidine_metabolism | 4.826 | 0.375 | 4.647 | 0.329 | 4.945 | 0.133 | 5.710 | 0.470 | 5.077 | 0.324 | 5.905 | 0.386 | 4.832 | 0.186 | 4.964 | 0.100 | 6.082 | 0.273 | 0.000 | 0.000 |
| ko00750: Vitamin_B6_metabolism | 0.554 | 0.071 | 0.531 | 0.103 | 0.505 | 0.039 | 0.453 | 0.053 | 0.644 | 0.094 | 0.426 | 0.049 | 0.651 | 0.160 | 0.664 | 0.076 | 0.396 | 0.035 | 0.000 | 0.000 |
| ko00670: One_carbon_pool_by_folate | 1.050 | 0.063 | 1.121 | 0.286 | 0.964 | 0.062 | 1.254 | 0.175 | 1.468 | 0.227 | 1.071 | 0.067 | 1.735 | 0.167 | 1.566 | 0.196 | 1.242 | 0.150 | 0.000 | 0.000 |
| ko00130: Ubiquinone_and_other_terpenoid_quinone_biosynthesis | 0.258 | 0.145 | 0.514 | 0.292 | 0.169 | 0.112 | 0.113 | 0.052 | 0.567 | 0.179 | 0.212 | 0.120 | 0.880 | 0.245 | 0.688 | 0.180 | 0.304 | 0.129 | 0.000 | 0.000 |
| ko00920: Sulfur_metabolism | 0.451 | 0.078 | 0.630 | 0.343 | 0.317 | 0.103 | 0.267 | 0.064 | 0.822 | 0.276 | 0.331 | 0.111 | 0.625 | 0.137 | 0.986 | 0.248 | 0.355 | 0.046 | 0.000 | 0.000 |
| ko00600: Sphingolipid_metabolism | 0.108 | 0.025 | 0.208 | 0.175 | 0.073 | 0.028 | 0.071 | 0.035 | 0.330 | 0.174 | 0.081 | 0.037 | 1.125 | 0.266 | 0.438 | 0.149 | 0.292 | 0.247 | 0.000 | 0.000 |
| ko00524: Butirosin_and_neomycin_biosynthesis | 0.069 | 0.009 | 0.128 | 0.097 | 0.064 | 0.015 | 0.126 | 0.019 | 0.240 | 0.084 | 0.065 | 0.012 | 0.257 | 0.052 | 0.285 | 0.090 | 0.096 | 0.045 | 0.000 | 0.000 |
| ko00410: beta_Alanine_metabolism | 0.254 | 0.085 | 0.357 | 0.230 | 0.140 | 0.057 | 0.638 | 0.275 | 0.626 | 0.185 | 0.280 | 0.071 | 0.668 | 0.088 | 0.658 | 0.143 | 0.458 | 0.124 | 0.000 | 0.000 |
| ko00626: Naphthalene_degradation | 0.130 | 0.027 | 0.108 | 0.069 | 0.108 | 0.043 | 0.096 | 0.029 | 0.038 | 0.015 | 0.157 | 0.047 | 0.035 | 0.021 | 0.040 | 0.012 | 0.161 | 0.055 | 0.000 | 0.000 |
| ko00908: Zeatin_biosynthesis | 0.017 | 0.008 | 0.066 | 0.088 | 0.011 | 0.006 | 0.014 | 0.011 | 0.162 | 0.090 | 0.037 | 0.026 | 0.128 | 0.055 | 0.207 | 0.083 | 0.060 | 0.035 | 0.000 | 0.000 |
| ko00730: Thiamine_metabolism | 0.661 | 0.052 | 0.906 | 0.386 | 0.580 | 0.169 | 0.481 | 0.080 | 1.038 | 0.167 | 0.505 | 0.127 | 0.423 | 0.171 | 1.142 | 0.196 | 0.460 | 0.107 | 0.000 | 0.000 |
| ko00622: Xylene_degradation | 0.117 | 0.028 | 0.108 | 0.068 | 0.103 | 0.048 | 0.068 | 0.020 | 0.028 | 0.008 | 0.111 | 0.015 | 0.029 | 0.030 | 0.025 | 0.015 | 0.105 | 0.029 | 0.000 | 0.000 |
| ko00020: Citrate_cycle_TCA_cycle_ | 1.410 | 0.125 | 1.608 | 0.234 | 1.271 | 0.100 | 2.070 | 0.463 | 2.031 | 0.287 | 1.623 | 0.199 | 1.637 | 0.185 | 2.048 | 0.194 | 1.863 | 0.221 | 0.000 | 0.000 |
| ko00280: Valine_leucine_and_isoleucine_degradation | 0.479 | 0.075 | 0.621 | 0.125 | 0.478 | 0.068 | 0.769 | 0.144 | 0.814 | 0.106 | 0.556 | 0.083 | 0.601 | 0.092 | 0.801 | 0.058 | 0.603 | 0.130 | 0.000 | 0.000 |
| ko00430: Taurine_and_hypotaurine_metabolism | 0.507 | 0.061 | 0.534 | 0.102 | 0.504 | 0.053 | 0.593 | 0.058 | 0.688 | 0.041 | 0.569 | 0.085 | 0.454 | 0.075 | 0.689 | 0.051 | 0.563 | 0.040 | 0.000 | 0.000 |
| ko00780: Biotin_metabolism | 0.103 | 0.034 | 0.173 | 0.106 | 0.059 | 0.044 | 0.042 | 0.023 | 0.194 | 0.047 | 0.060 | 0.043 | 0.149 | 0.033 | 0.223 | 0.056 | 0.063 | 0.015 | 0.000 | 0.001 |
| ko00625: Chloroalkane_and_chloroalkene_degradation | 0.154 | 0.030 | 0.114 | 0.063 | 0.126 | 0.046 | 0.132 | 0.042 | 0.052 | 0.033 | 0.156 | 0.035 | 0.045 | 0.026 | 0.042 | 0.020 | 0.154 | 0.051 | 0.000 | 0.001 |
| ko00460: Cyanoamino_acid_metabolism | 0.455 | 0.095 | 0.554 | 0.173 | 0.420 | 0.019 | 0.467 | 0.056 | 0.731 | 0.137 | 0.465 | 0.041 | 0.591 | 0.089 | 0.793 | 0.148 | 0.482 | 0.078 | 0.000 | 0.001 |
| ko00940: Phenylpropanoid_biosynthesis | 0.030 | 0.036 | 0.065 | 0.076 | 0.034 | 0.038 | 0.020 | 0.037 | 0.133 | 0.088 | 0.047 | 0.021 | 0.242 | 0.076 | 0.171 | 0.075 | 0.076 | 0.053 | 0.000 | 0.001 |
| ko00362: Benzoate_degradation | 0.532 | 0.068 | 0.583 | 0.177 | 0.474 | 0.063 | 0.398 | 0.069 | 0.405 | 0.052 | 0.558 | 0.110 | 0.232 | 0.081 | 0.386 | 0.045 | 0.439 | 0.109 | 0.000 | 0.001 |
| ko00440: Phosphonate_and_phosphinate_metabolism | 0.029 | 0.008 | 0.091 | 0.105 | 0.018 | 0.018 | 0.009 | 0.010 | 0.181 | 0.088 | 0.022 | 0.018 | 0.037 | 0.047 | 0.232 | 0.085 | 0.012 | 0.009 | 0.000 | 0.001 |
| ko00540: Lipopolysaccharide_biosynthesis | 0.187 | 0.098 | 0.449 | 0.356 | 0.117 | 0.108 | 0.336 | 0.144 | 0.468 | 0.169 | 0.202 | 0.099 | 1.068 | 0.149 | 0.538 | 0.127 | 0.398 | 0.239 | 0.000 | 0.001 |
| ko01051: Biosynthesis_of_ansamycins | 0.178 | 0.043 | 0.106 | 0.098 | 0.147 | 0.085 | 0.157 | 0.038 | 0.046 | 0.033 | 0.114 | 0.038 | 0.218 | 0.041 | 0.048 | 0.025 | 0.157 | 0.042 | 0.000 | 0.001 |
| ko00720: Carbon_fixation_pathways_in_prokaryotes | 2.260 | 0.176 | 2.369 | 0.213 | 2.086 | 0.130 | 3.004 | 0.560 | 2.875 | 0.320 | 2.440 | 0.266 | 2.340 | 0.228 | 2.755 | 0.155 | 2.551 | 0.249 | 0.000 | 0.002 |
| ko00564: Glycerophospholipid_metabolism | 0.778 | 0.141 | 0.806 | 0.261 | 0.593 | 0.164 | 0.588 | 0.100 | 0.520 | 0.080 | 0.756 | 0.343 | 1.898 | 0.365 | 0.557 | 0.064 | 1.032 | 0.357 | 0.000 | 0.002 |
| ko00620: Pyruvate_metabolism | 3.221 | 0.107 | 2.954 | 0.283 | 3.074 | 0.169 | 3.183 | 0.241 | 2.839 | 0.241 | 3.204 | 0.111 | 2.471 | 0.259 | 2.705 | 0.164 | 3.116 | 0.193 | 0.000 | 0.002 |
| ko00471: D_Glutamine_and_D_glutamate_metabolism | 0.314 | 0.053 | 0.345 | 0.169 | 0.217 | 0.045 | 0.305 | 0.058 | 0.482 | 0.142 | 0.250 | 0.097 | 0.540 | 0.093 | 0.564 | 0.142 | 0.332 | 0.078 | 0.000 | 0.002 |
| ko00120: Primary_bile_acid_biosynthesis | 0.022 | 0.016 | 0.013 | 0.019 | 0.017 | 0.010 | 0.013 | 0.011 | 0.007 | 0.006 | 0.046 | 0.050 | 0.236 | 0.059 | 0.018 | 0.022 | 0.090 | 0.064 | 0.000 | 0.002 |
| ko00121: Secondary_bile_acid_biosynthesis | 0.022 | 0.016 | 0.013 | 0.019 | 0.017 | 0.010 | 0.013 | 0.011 | 0.007 | 0.006 | 0.046 | 0.050 | 0.236 | 0.059 | 0.018 | 0.022 | 0.090 | 0.064 | 0.000 | 0.002 |
| ko01055: Biosynthesis_of_vancomycin_group_antibiotics | 0.296 | 0.057 | 0.258 | 0.164 | 0.359 | 0.048 | 0.285 | 0.039 | 0.172 | 0.069 | 0.326 | 0.077 | 0.162 | 0.044 | 0.130 | 0.094 | 0.287 | 0.059 | 0.000 | 0.002 |
| ko00960: Tropane_piperidine_and_pyridine_alkaloid_biosynthesis | 0.151 | 0.025 | 0.112 | 0.027 | 0.114 | 0.019 | 0.106 | 0.015 | 0.112 | 0.019 | 0.075 | 0.028 | 0.184 | 0.084 | 0.118 | 0.009 | 0.081 | 0.026 | 0.000 | 0.003 |
| ko00500: Starch_and_sucrose_metabolism | 2.579 | 0.266 | 2.539 | 0.344 | 3.001 | 0.241 | 2.457 | 0.272 | 2.297 | 0.205 | 2.897 | 0.214 | 2.652 | 0.196 | 2.202 | 0.260 | 2.872 | 0.156 | 0.000 | 0.003 |
| ko00480: Glutathione_metabolism | 0.576 | 0.052 | 0.717 | 0.215 | 0.515 | 0.090 | 0.541 | 0.025 | 0.761 | 0.095 | 0.593 | 0.108 | 0.431 | 0.112 | 0.842 | 0.073 | 0.580 | 0.128 | 0.000 | 0.003 |
| ko00052: Galactose_metabolism | 2.147 | 0.132 | 2.173 | 0.375 | 2.593 | 0.289 | 2.024 | 0.242 | 2.042 | 0.211 | 2.378 | 0.102 | 2.656 | 0.182 | 1.944 | 0.269 | 2.363 | 0.181 | 0.000 | 0.003 |
| ko00270: Cysteine_and_methionine_metabolism | 2.916 | 0.213 | 2.750 | 0.414 | 2.839 | 0.352 | 2.469 | 0.224 | 2.387 | 0.184 | 2.645 | 0.099 | 1.909 | 0.206 | 2.466 | 0.143 | 2.527 | 0.308 | 0.000 | 0.003 |
| ko00590: Arachidonic_acid_metabolism | 0.017 | 0.015 | 0.056 | 0.049 | 0.012 | 0.012 | 0.006 | 0.004 | 0.079 | 0.042 | 0.011 | 0.009 | 0.019 | 0.022 | 0.100 | 0.035 | 0.007 | 0.006 | 0.000 | 0.004 |
| ko00010: Glycolysis_Gluconeogenesis | 4.330 | 0.379 | 4.397 | 0.875 | 5.132 | 0.430 | 4.611 | 0.211 | 4.281 | 0.434 | 4.971 | 0.308 | 2.995 | 0.441 | 4.108 | 0.519 | 4.659 | 0.524 | 0.000 | 0.004 |
| ko00030: Pentose_phosphate_pathway | 3.954 | 0.422 | 3.667 | 1.125 | 4.690 | 0.621 | 3.932 | 0.372 | 3.234 | 0.753 | 4.290 | 0.449 | 2.379 | 0.499 | 2.782 | 0.789 | 3.837 | 0.523 | 0.000 | 0.004 |
| ko00550: Peptidoglycan_biosynthesis | 1.144 | 0.095 | 1.140 | 0.324 | 0.786 | 0.148 | 1.005 | 0.102 | 1.311 | 0.234 | 1.042 | 0.362 | 1.742 | 0.284 | 1.449 | 0.281 | 1.219 | 0.257 | 0.000 | 0.005 |
| ko00051: Fructose_and_mannose_metabolism | 2.757 | 0.303 | 2.913 | 0.102 | 2.975 | 0.092 | 2.495 | 0.235 | 2.758 | 0.206 | 3.001 | 0.241 | 2.202 | 0.301 | 2.853 | 0.122 | 2.886 | 0.229 | 0.000 | 0.005 |
| ko00643: Styrene_degradation | 0.077 | 0.012 | 0.069 | 0.048 | 0.066 | 0.018 | 0.060 | 0.024 | 0.027 | 0.021 | 0.069 | 0.031 | 0.021 | 0.013 | 0.026 | 0.022 | 0.042 | 0.007 | 0.000 | 0.005 |
| ko00740: Riboflavin_metabolism | 0.163 | 0.044 | 0.282 | 0.121 | 0.128 | 0.057 | 0.222 | 0.045 | 0.264 | 0.041 | 0.218 | 0.118 | 0.400 | 0.088 | 0.303 | 0.040 | 0.293 | 0.083 | 0.000 | 0.006 |
| ko00633: Nitrotoluene_degradation | 0.294 | 0.053 | 0.266 | 0.165 | 0.233 | 0.075 | 0.191 | 0.038 | 0.097 | 0.081 | 0.182 | 0.058 | 0.081 | 0.054 | 0.081 | 0.055 | 0.205 | 0.096 | 0.000 | 0.007 |
| ko00250: Alanine_aspartate_and_glutamate_metabolism | 3.902 | 0.369 | 3.467 | 0.563 | 3.859 | 0.251 | 3.935 | 0.255 | 3.483 | 0.323 | 3.887 | 0.275 | 2.726 | 0.299 | 3.362 | 0.278 | 3.675 | 0.339 | 0.000 | 0.007 |
| ko00072: Synthesis_and_degradation_of_ketone_bodies | 0.113 | 0.032 | 0.109 | 0.085 | 0.076 | 0.023 | 0.092 | 0.015 | 0.037 | 0.015 | 0.108 | 0.042 | 0.039 | 0.023 | 0.045 | 0.018 | 0.093 | 0.036 | 0.000 | 0.008 |
| ko00450: Selenocompound_metabolism | 0.764 | 0.074 | 0.844 | 0.220 | 0.628 | 0.100 | 0.773 | 0.073 | 1.023 | 0.188 | 0.751 | 0.035 | 0.739 | 0.110 | 1.150 | 0.173 | 0.769 | 0.051 | 0.000 | 0.008 |
| ko00650: Butanoate_metabolism | 1.581 | 0.117 | 1.587 | 0.203 | 1.376 | 0.132 | 1.521 | 0.127 | 1.371 | 0.134 | 1.504 | 0.118 | 1.190 | 0.150 | 1.264 | 0.108 | 1.402 | 0.091 | 0.000 | 0.010 |
| ko00071: Fatty_acid_metabolism | 0.303 | 0.057 | 0.331 | 0.163 | 0.210 | 0.063 | 0.350 | 0.070 | 0.193 | 0.024 | 0.295 | 0.030 | 0.304 | 0.063 | 0.203 | 0.017 | 0.337 | 0.043 | 0.000 | 0.011 |
| ko00760: Nicotinate_and_nicotinamide_metabolism | 0.858 | 0.167 | 1.008 | 0.257 | 0.760 | 0.155 | 0.893 | 0.146 | 1.122 | 0.133 | 0.888 | 0.168 | 1.141 | 0.078 | 1.182 | 0.164 | 0.948 | 0.116 | 0.000 | 0.016 |
| ko00253: Tetracycline_biosynthesis | 0.241 | 0.045 | 0.211 | 0.090 | 0.143 | 0.057 | 0.136 | 0.031 | 0.157 | 0.051 | 0.152 | 0.049 | 0.073 | 0.037 | 0.161 | 0.028 | 0.127 | 0.048 | 0.000 | 0.028 |
| ko00040: Pentose_and_glucuronate_interconversions | 2.499 | 0.519 | 2.267 | 0.517 | 2.884 | 0.468 | 2.187 | 0.406 | 1.931 | 0.461 | 2.431 | 0.393 | 1.467 | 0.355 | 1.637 | 0.558 | 1.984 | 0.522 | 0.000 | 0.032 |
| ko00565: Ether_lipid_metabolism | 0.000 | 0.000 | 0.000 | 0.000 | 0.000 | 0.000 | 0.001 | 0.002 | 0.000 | 0.000 | 0.001 | 0.003 | 0.002 | 0.002 | 0.000 | 0.000 | 0.002 | 0.002 | 0.000 | 0.039 |
| ko00312: beta_Lactam_resistance | 0.018 | 0.009 | 0.017 | 0.013 | 0.008 | 0.009 | 0.006 | 0.006 | 0.002 | 0.001 | 0.011 | 0.010 | 0.003 | 0.004 | 0.001 | 0.001 | 0.005 | 0.003 | 0.000 | 0.042 |

Table 3S. Highest scoring signals in each NMR spectral latent variable that passed feature selection in at least one cluster map. These groups of signals can be seen as the patterns responsible for best treatment group separation in each latent variable.

| Latent Variable | Metabolite | Group of Signals (shift in ppm) | Kruskal p-value at T0 | Kruskal p-value at T1 | Kruskal p-value at T2 |
| --- | --- | --- | --- | --- | --- |
| LV0 | Myo-Inositol | 3.560:3.451 | 0.850582 | 2.490724e-05 | 0.020061 |
|  | Acetate | 2.008:1.935 | 0.051943 | 0.013601 | 9.5833612e-05 |
|  | Oxo-Iso Valerate | 1.149:1.095 | 0.177288 | 0.002629 | 0.84375 |
| LV2 | Formate | 8.480:8.425 | 0.511901 | 0.997214 | 0.9924374 |
|  | Hypoxanthine | 8.224:8.151 | 0.901121 | 0.801032 | 0.001709 |
|  | Creatine Phosphate | 3.067:3.031 | 0.10698 | 0.063588 | 0.0019111 |
|  | Trimethylamine | 2.903:2.866 | 0.56174 | 0.0808736 | 5.070378e-05 |
|  | Butyrate/butanoate/butanoic acid | 1.570:1.533, 0.912:0.857 | 0.376810 | 0.029469 | 0.0002058 |
|  | Alanine | 1.497:1.442 | 0.710756 | 4.1426e-08 | 0.0247327 |
| LV3 | Urocanate | 7.950:7.804 | 0.004877 | 0.519259 | 0.004132 |
|  | Trimethylamine | 2.903:2.866 | 0.56174 | 0.0808736 | 5.070378e-05 |
|  | Oxo-Iso Valerate | 1.149:1.095 | 0.177288 | 0.002629 | 0.84375 |
| LV4 | Nicotinate | 8.315:8.242 | 0.60871 | 0.33954 | 0.06010 |
|  | Hypoxanthine | 8.224:8.151 | 0.901121 | 0.801032 | 0.001709 |
|  | Dihydroxyacetone | 4.474:4.382 | 0.012191 | 0.11853 | 0.88664 |
|  | Glutamate | 2.373:2.337, 2.136:2.008 | 0.011699 | 0.050324 | 0.00556 |
| LV5 | Nicotinate | 8.315:8.242 | 0.60871 | 0.33954 | 0.06010 |
|  | Glutamate | 2.373:2.337, 2.136:2.008 | 0.011699 | 0.050324 | 0.00556 |
|  | Acetate | 2.008:1.935 | 0.051943 | 0.013601 | 9.5833612e-05 |
|  | Propanoate/  propionic acid | 1.076:1.003 | 0.71868 | 0.53726 | 0.014673 |
| LV6 | Nicotinate | 8.315:8.242 | 0.60871 | 0.33954 | 0.06010 |
|  | Tyrosine | 7.128:6.818 | 0.009453 | 0.00015 | 0.30657 |
|  | Lactate | 4.182:4.090, 1.369:1.314 | 0.60045 | 0.024294 | 0.00061 |
|  | Glutamate | 2.373:2.337, 2.136:2.008 | 0.011699 | 0.050324 | 0.00556 |
| LV9 | Nicotinate | 8.315:8.242 | 0.60871 | 0.33954 | 0.06010 |
|  | Lactate | 4.182:4.090, 1.369:1.314 | 0.60045 | 0.024294 | 0.00061 |

Table 4S. Correlation of bacterial species from shotgun metagenomics with PCoA axes

| **Bacteria species** | **axis** | **R** | **p.value** |
| --- | --- | --- | --- |
| *Pseudoflavonifractor_capillosus* | MDS1 | -0.740 | <0.000 |
| *Ruminococcus_torques* | MDS1 | -0.723 | <0.000 |
| *Bacteroides_fragilis* | MDS1 | 0.703 | <0.000 |
| *Oscillibacter_unclassified* | MDS1 | -0.556 | <0.000 |
| *Alistipes_unclassified* | MDS1 | 0.531 | <0.000 |
| *Anaerotruncus_colihominis* | MDS1 | -0.510 | <0.000 |
| *Subdoligranulum_spp* | MDS1 | -0.484 | <0.000 |
| *Erysipelotrichaceae_bacterium_21_3* | MDS1 | -0.428 | <0.000 |
| *Lachnospiraceae_bacterium_1_1_57FAA* | MDS1 | -0.420 | <0.000 |
| *Lachnospiraceae_bacterium_8_1_57FAA* | MDS1 | -0.345 | <0.006 |
| *Faecalibacterium_prausnitzii* | MDS1 | -0.343 | <0.006 |
| *Escherichia_coli* | MDS1 | -0.338 | <0.007 |
| *Alistipes_spp* | MDS2 | -0.826 | <0.000 |
| *Bacteroides_fragilis* | MDS2 | 0.688 | <0.000 |
| *Lactobacillus_crispatus* | MDS3 | -0.741 | <0.000 |
| *Lactobacillus_salivarius* | MDS3 | -0.697 | <0.000 |
| *Lactobacillus_johnsonii* | MDS3 | -0.627 | <0.000 |
| *Anaerotruncus_colihominis* | MDS3 | 0.605 | <0.000 |
| *Lactobacillus_reuteri* | MDS3 | -0.478 | <0.000 |
| *Lachnospiraceae_bacterium_1_1_57FAA* | MDS3 | -0.429 | <0.000 |
| *Subdoligranulum_variabile* | MDS3 | -0.345 | <0.006 |
